# Protocol to visualize a bodily map of musical uncertainty and prediction

**DOI:** 10.1016/j.xpro.2024.103473

**Published:** 2024-12-05

**Authors:** Tatsuya Daikoku, Masaki Tanaka

**Affiliations:** 1 Graduate School of Information Science and Technology, The University of Tokyo, Tokyo, Japan

**Keywords:** neuroscience, cognitive neuroscience, behavior

## Abstract

Music deeply influences our bodies as well as minds. Here, we present a protocol to visualize a bodily sensation map elicited by music perception using body-mapping tests and emotional evaluations. We describe steps for setting the body-map test and musical stimuli based on prediction. We then detail procedures for visualizing the body map and identifying body areas in which musical stimuli influence subjective sensations.

For complete details on the use and execution of this protocol, please refer to Daikoku et al.^1^

## Highlights

• Generate a predictive model of musical chords by HBSL model
• Participants map sensations after listening to chord progressions
• Interpolate coordinates of x and y in a mesh grid format with a color map
• Generate bodily sensation maps of music prediction

## Abstract (editor highlights)

Publisher’s note: Undertaking any experimental protocol requires adherence to local institutional guidelines for laboratory safety and ethics.

## Graphical abstract

**Figure:**
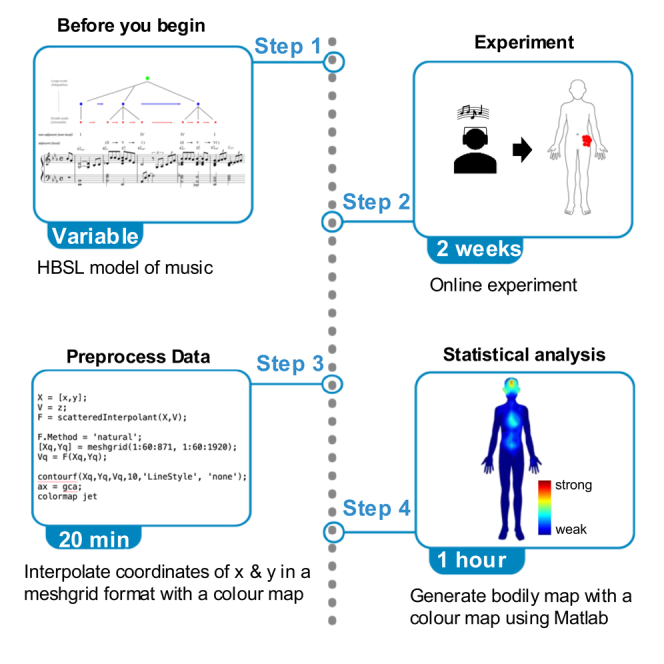

## Before you begin

### Participants

1. Ensure ethical approval is obtained for participant involvement.
2. Prepare consent forms and explain the study to participants.
3. Get their informed consent from participants before the experiment.

### Institutional permissions

This study protocol was approved by the Ethics Committee of The University of Tokyo (Screening number: 21–335) and conducted in accordance with the guidelines of the Declaration of Helsinki.

### Design experimental task

**Timing: variable**

4. To generate a predictive model of musical chord progression, use a Hierarchical Bayesian Statistical Learning (HBSL) Model.^2^^,^^3^
  a. Prepare a corpus of 890 pop songs in the McGill Billboard Corpus (https://ddmal.music.mcgill.ca/research/The_McGill_Billboard_Project_(Chord_Analysis_Dataset)/).^4^
  b. Download the package of HBSL model (to download: https://osf.io/zjwxe/?view_only=4a2f14edd00c4ca391d8befe2e646c73).^2^^,^^3^ The detailed instruction was described in our paper.^1^
  c. Put the corpus data in the folder “corpus” of the package of HBSL model.
  d. Describe all of the file names (including extension) of music samples in the file “corpusList.csv”
  e. Set the parameters from the PreSet.txt of the package of HBSL model.
    i. “n” of the first line represents n-gram. Here, set n = 2.
    ii. “type1” of the second line represents whether the model learns absolute or relative pitch. Here, set type1 = ab_.
    iii. “type2” of the third line represents whether the model learns only highest-pitched sequence (≒ melody) or all tone sequences. Here, set type2 = all_tone because the model learns chords having concurrent multiple tones.
    iv. “chunk_constant” of the fourth line represents whether the model chunks sequential stimuli by statistical learning or not, and how easily the model chunks. Here, set chunk_constant = 0 because the model does not need to chunk given the purpose of this study.
    v. The other parameters are not used in this study.
  f. Execute the python’s script “HSL.py” to generate a predictive model of musical chord progression.
  g. Execute the python’s script “Melody_generator.py” to generate the chord progressions encompassed within the different 8 types of chord progressions, which are characterized by varying degrees of uncertainty and surprise.^1^
  h. The composed chord progressions are generated in the directory of result/composition/. The generated music samples are deposited to an external source (https://osf.io/cyqhd/).
5. Use Gorilla Experiment Builder (https://gorilla.sc), which is a cloud-based research platform that allows the deploying of behavioral experiments online.
  a. Download the body image template (body.png) from the external source (https://osf.io/rs4kh/).^5^
  b. Use the body image in the body map test after listening each of the composed chord progression.

## Key resources table

**Table:** 

REAGENT or RESOURCE	SOURCE	IDENTIFIER
**Software and algorithms**		

Body-mapping software	Custom	N/A
Emotional evaluation forms	Custom	N/A
Statistical-learning model	Open-source	https://example.com/model
MATLAB 2022b	https://jp.mathworks.com/products/matlab.html	RRID:SCR_001622
GORILLA Experiment Builder	https://gorilla.sc/	RRID:SCR_020991; https://app.gorilla.sc/openmaterials/898370
Python 3.1.1	www.python.org	RRID:SCR_008394

## Step-by-step method details

### Perform experiment

**Timing: 2 weeks**

**Figure 1:**
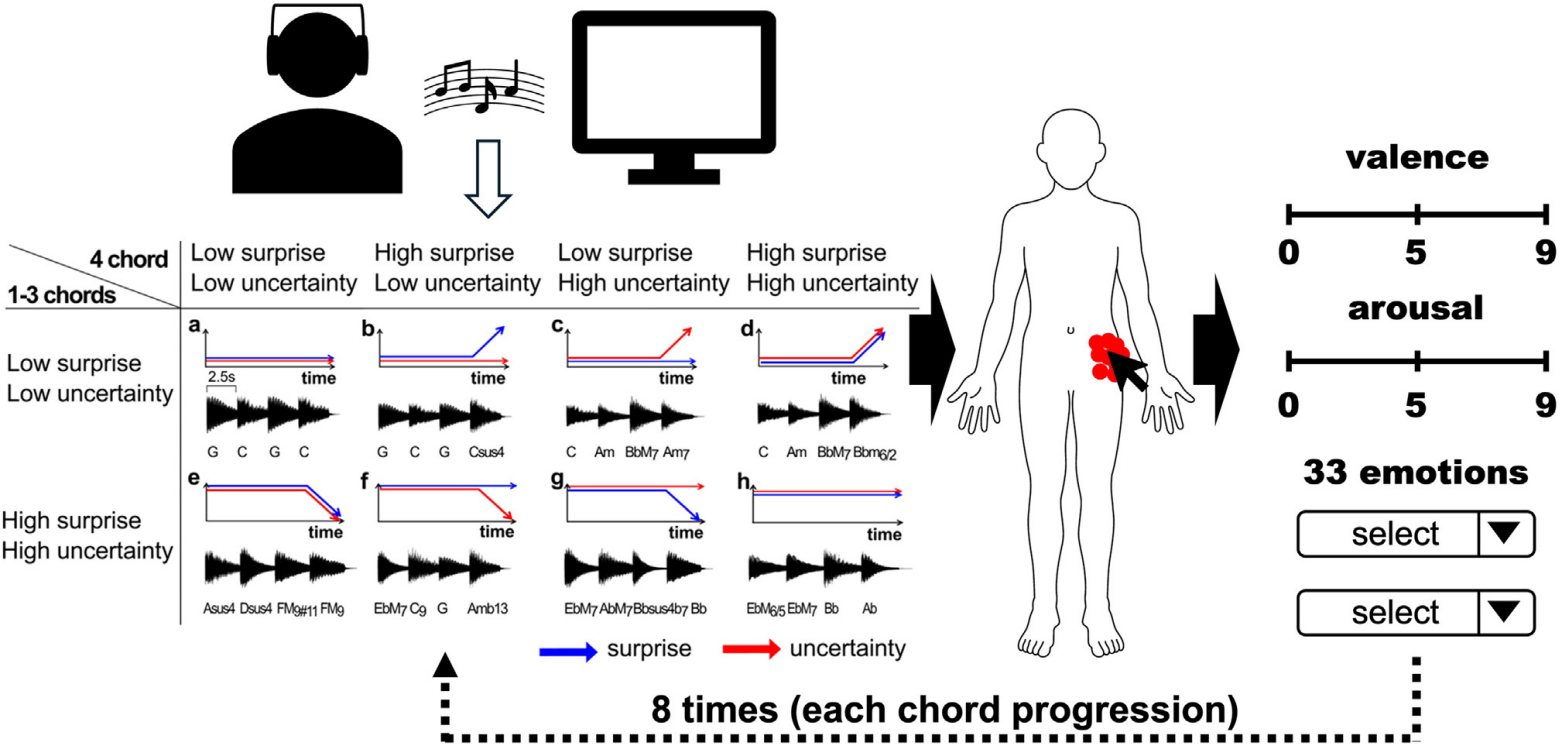
Overview Experimental protocol, reprinted from Tanaka and Daikoku, 2024 Below are 8 types of chord progressions. The blue and red arrows indicate surprise and uncertainty values, respectively.^8^

This section describes how to perform the experiment (Figure 1).

1. Present the eight types of the stimuli in random order to the participants.
2. In each eight types of the stimuli, ask them to respond within 100 times with clicks to the position in the body where they felt sensations from the chords, using the body image presented on the screen.
3. Ask the following two types of surveys to obtain emotional judgments.
  a. Multiple-choice categorical judgments; that is, in each type of chord progression, participants were required to select the best 5 emotional categories in ranking elicited by each sound from a list of 33 categories (see in the supplementary material).
  b. Nine-point dimensional judgments; that is, after hearing the chord progression, participants were required to rate each type of chord progression along the valence and arousal. Each rating was obtained on a nine-point Likert scale with the number 5 anchored at neutral.

### Preprocess data

**Timing: 20 min**

This section describes how to preprocess the recorded data.

4. Download the coordinate data of x and y in the body mapping test and the data of answers of emotion survey from the account of Gorilla Experiment Builder (https://gorilla.sc).
5. Interpolate the coordinates of x and y in a mesh grid format with a color map that represented the neighboring points.

### Statistical analysis

**Timing: 1 h**

This section describes how to perform statistical analysis of the preprocessed data.

6. Down-sample the raw data of x and y coordinates (The body image template has a pixel size of 871 pixels in width and 1920 pixels in height) by a factor of 40.
7. Generate the bodily map with a color map using a function of “contourf” of Matlab (2022b).
8. Extract the total number of clicks at two interoceptive positions including cardiac and abdomen areas in each participant, using the coordinate data of x and y in the body mapping test.
  a. Demarcate specific regions to represent distinct bodily areas.
    ***Note:*** Here, the cardiac region spanned a width from 360 to 550 pixels and a height from 390 to 620 pixels, while the abdominal region was delineated between 360 to 510 pixels in width and 650 to 850 pixels in height.
    ***Note:*** The click data may exhibit dependencies between sensations in one area and those in others both in terms of spatial autocorrelation, where individuals may indicate sensations over large areas, and in terms of individual differences, where individuals vary in the overall intensity or amount of sensation they report. The intensity is calculated based on the number of clicks in the same body position.
  b. Conduct a generalized linear mixed model analysis (GLMM) to assess the individual differences and dependencies between total clicks and those associated with the cardiac, abdominal, and head regions, as well as the relationship between cardiac and abdominal clicks The example of model formula is as follows:
    Abdomen∼Heart+(1|Subjects)
    In this context, 'abdomen' is the response variable, 'heart' acts as the explanatory variable, and 'subjects' represent random effects. The model considers the dependency of 'abdomen' on 'heart' while addressing the correlation structure within data points from the same subjects. The notation (1∣subjects)(1∣subjects) signifies unique random effects for each subject, accommodating individual variability. This framework ensures the model accurately captures correlations among observations within each individual.
  c. Perform the Shapiro–Wilk test for normality on the total number of clicks at cardiac, abdomen, and head positions in each participant.
    i. Based on the test results, apply either parametric or non-parametric (Friedman) repeated-measures analysis of variance (ANOVA) to compare these click counts, as well as the scores for valence, arousal, and categorical emotional responses across different types of chord progressions.
      ***Note:*** The dependent variables include the total clicks at each body area and the emotional scores, while the within-subject factor was the eight types of chord progressions.
9. Use the results of the best 5 emotional categories in the ranking to score the intensity of 33 emotions.
  a. Score the first, second, third, fourth, and fifth categories as a 5, 4, 3, 2, and 1 point respectively.
  b. Average the scores of each 33 emotional categories for all participants.
  c. Perform the Shapiro–Wilk test for normality on the 33 emotional scores of the multiple-choice categorical judgments, and the valence and arousal scores of the nine-point dimensional judgments.
    i. Depending on the result of the test for normality, either the parametric or non-parametric (Friedman) repeated-measure analyses of variance (Friedman’s ANOVA) were applied to compare the total number of clicks at cardiac, abdomen, and head positions, and the scores of valence, arousal, and categorical emotional scores, among different types of chord progressions.

***Note:*** The dependent variable was the total number of clicks for each cardiac, abdomen, and head area and the scores of valence, arousal, and categorical emotional scores, and the within-subject factor was the 8 types of chord progressions.

## Expected outcomes

After performing all analyses described above, the expected outcome is that the bodily sensation will be predominantly concentrated in the heart and abdominal regions.^1^

## Limitations

There is variation in the number of chord progressions within each of the eight groups. To address the potential bias stemming from this variability, we balanced the dataset by considering the ratio of participants' responses within each group. This balancing approach helps address the issue of unequal data distribution and enhances the statistical robustness of our analysis. However, it is important to acknowledge that we cannot entirely eliminate the possibility of bias affecting the results. Also, current body-map studies do not directly investigate physical reaction such as electrocardiography, electrogastrography elicited by music perception. That is, the experimental paradigm based on a questionnaire (e.g., having participants click on a bodily map) may not adequately capture the nuances of interoceptive experiences. While it might not offer the granularity of more specialized interoceptive tasks, it serves as a bridge between subjective experiences and more objective measures.^6^^,^^7^

## Troubleshooting

### Problem 1

The location of the heart and abdomen is determined subjectively by the analyst. Moreover, individuals with a right-sided heart position may have differing perceptions of their cardiac sensation.

### Potential solution

• A generalized body map can be created using the results of body mapping from all participants. Then, by confirming the overall location of cardiac sensation based on this map, the position of the heart can be determined.
• Following the body mapping test, ask participants to provide narrative responses explaining why they clicked in specific locations to understand whether clicks are truly indicative of cardiac sensation.

### Problem 2

The raw click data (a pixel size of 871 pixels in width and 1920 pixels in height) of body map test is downsampled by dividing into 40-pixel increments. However, determining the appropriate degree of downsampling computationally is necessary.

### Potential solution

Instead of using images such as those with a pixel size of 871 pixels in width and 1920 pixels in height, set a constraint from the outset where only one click is allowed within every 40 pixels.

## Resource availability

### Lead contact

Further information and requests for resources should be directed to and will be fulfilled by the lead contact, Tatsuya Daikoku (daikoku.tatsuya@mail.u-tokyo.ac.jp).

### Technical contact

Questions about the technical specifics of performing the protocol should be directed to and will be answered by the technical contact, Tatsuya Daikoku (daikoku.tatsuya@mail.u-tokyo.ac.jp).

### Materials availability

This study did not generate new unique reagents.

### Data and code availability

• All of the anonymized raw data files, stimuli used in this study, and the results of statistical analysis have been deposited to an external source (https://osf.io/cyqhd/) and are publicly available as of the date of publication. The other data are shown in supplementary data.
• This paper does not report original code.
• Any additional information required to reanalyze the data reported in this paper is available from the lead contact upon request.

## Acknowledgments

This research was supported by the Japan Science and Technology Agency (JST) Moonshot Goal 9 (JPMJMS2297) and JSPS KAKENHI (24H01539 and 24H00898), Japan.

## Author contributions

T.D. and M.T. designed the experimental task, developed analytical protocols, and wrote the paper.

## Declaration of interests

The authors declare no competing interests.
